# Mental Health Status of Medical Staff Exposed to Hospital Workplace Violence: A Prospective Cohort Study

**DOI:** 10.3389/fpubh.2022.930118

**Published:** 2022-07-12

**Authors:** Licheng Wang, Xin Ni, Zhe Li, Yuanshuo Ma, Yafeng Zhang, Zhong Zhang, Lei Gao, Xinyan Liu, Wenxin Yan, Lihua Fan, Lei Shi

**Affiliations:** ^1^School of Health Management, Harbin Medical University, Harbin, China; ^2^National Center for Children's Health, Children's Hospital Affiliated Capital Medical University, Beijing, China; ^3^The Fourth Affiliated Hospital of Harbin Medical University, Harbin, China; ^4^School of Health Management, Southern Medical University, Guangzhou, China

**Keywords:** medical staff, hospital workplace violence, mental health loss, hospital, a prospective cohort study

## Abstract

**Objective:**

Hospital workplace violence is one of the most frequently reported types of workplace violence in the world, and it harms the mental and physical health of medical staff. Existing research on workplace violence focused more on cross-sectional studies, than longitudinal studies. Therefore, this study examined the dynamic impact of hospital workplace violence on the mental health of medical staff, with the aim of providing appropriate countermeasures and suggestions for hospitals to develop targeted psychological intervention measures in time for medical staff who experience workplace violence.

**Design:**

A prospective cohort study.

**Participants:**

A total of 112 medical staff who had experienced workplace violence in the hospital for the first time were chosen.

**Setting:**

An unconditional latent variable growth curve model was established based on the average value of the general health scale scores of medical staff, and gender and violence types (intimidation threats, physical violence) as control variables. Each medical staff was assessed for depression and anxiety at 4 different time points and the changes in the trajectories was observed.

**Results:**

There were significant differences in the incidence of mental health and anxiety among medical staff at different observation times. There were significant individual differences in the initial mental health status, growth rate of anxiety and depression of medical staff.

**Conclusions:**

Hospitals should undertake various psychological intervention strategies based on the staff's mental health to safeguard those who have experienced workplace violence.

## Background

Hospital workplace violence has become a global public health concern and social issue (1–4). Thus, hospital workplace violence is neither incidental in some hospitals, nor is it confined to a single country or region; it is a pervasive problem in medical professions (5–7).

### Workplace Violence in Hospitals Results in Serious Harm to Society

Hospital workplace violence can result in the death of medical staff as well as the disruption of medical order. In a report of workplace violence occurrences publicized by the media of 310 cases between 2000 and 2018, there were 49.4% cases of light or minor injuries to medical staff, 21.3% serious injuries, 9.7% fatalities, and 7.4% cases of severe property damage to hospitals (8). Jia et al. (9), which surveyed national public hospitals in 2003–2014, showed that 84.0% of hospital management personnel, 78.0% of medical staff, and 51.2% of patients believed that malignant medical harm was a criminal offense in China.

Hospital workplace violence may also have other negative effects on health care workers, such as affecting self-esteem (10), job performance (11), job satisfaction (12), job stress (13), job burnout (12), and turnover intention (14).

It is noteworthy that hospital workplace violence could increase the risk of type 2 diabetes (15) and reduce the quality of life of health care workers (13). Violence in the hospital workplace can also trigger negative emotions among medical staff, including depression and anxiety, and may indirectly contribute to sleep disturbances (16). It may also enhance the risk of post-traumatic stress disorder correspondingly (15).

### Mental Health Loss

This study considers general health, anxiety, and depression status as indicators of mental health loss, and describes the current situation of mental health caused by hospital workplace violence.

To the authors' knowledge, there is very limited evidence of the changes in mental health of medical staff after being exposed to hospital workplace violence. Therefore, it is important for studies to bridge these gaps. In this regard, this study proposes an innovative longitudinal follow-up method for research on mental health loss of medical staff exposed to hospital workplace violence in China.

The purpose of this study was to explore the dynamic changes in psychological loss of medical staff after experiencing hospital workplace violence, as well as to provide scientific guidance to hospitals in developing appropriate psychological intervention measures which can contribute to maintaining the stability of human resources.

## Methods

### Sample and Participants

A prospective cohort study was conducted from June 2017 to December 2018 in nine tertiary hospitals in Beijing and Heilongjiang Province, China, using a purposive sampling method. The target sample was medical staff suffering from hospital workplace violence for the first time. The observation time was 6 months, and the participants were assessed 7 days, 1, 3, and 6 months after they enrolled in the study.

**Figure 1:**
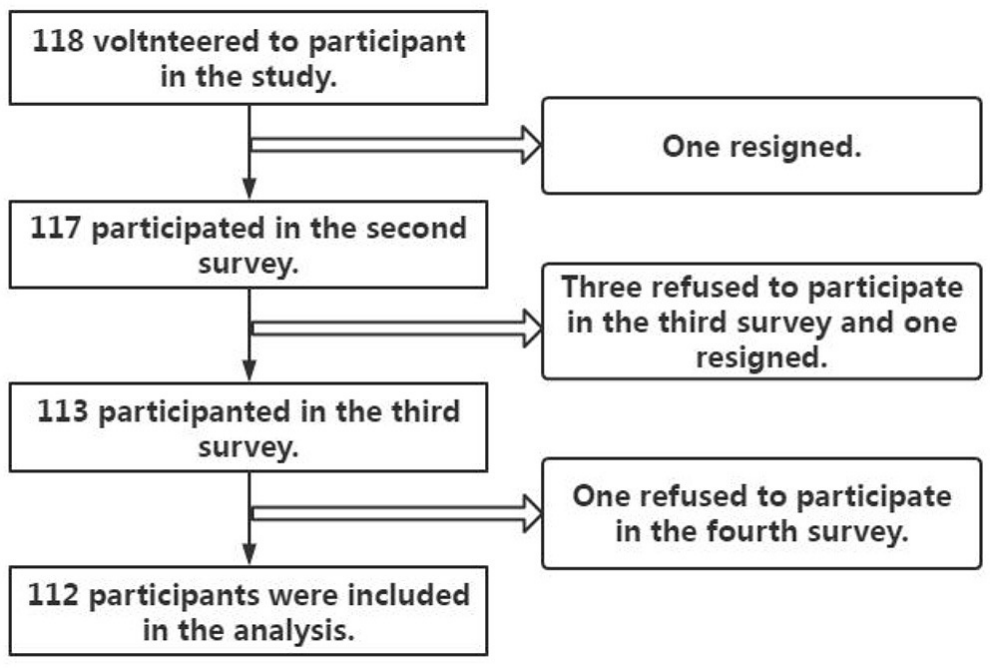
Flowchart of the medical staff selection.

The required sample size of medical staff calculated by PASS software was 85. Considering the loss of follow-up and elimination of subjects, the sample size was increased by 30%, reaching a proposed sample size of about 111 people. A total of 118 participants who had experienced workplace violence in the hospital for the first time were chosen. Six were lost to follow-up, of this six person, two resigned, three refused to participate in the third survey, and one refused to participate in the fourth survey. Thus, the data of 112 were included in the final analysis, as shown in Figure 1.

Subjects were included in the study if they met the following inclusion criteria: medical staff who had been subjected to intimidation, threats, or physical assault for the first time; and no recurrence of violence, other life events, or other traumatic events in the past 6 months or during follow-up. Individuals were excluded if they had family inheritance or a history of mental illness or were lost to follow-up. All medical staff participating in this study provided informed consent and participated voluntarily.

### Assessment Tools

#### Demographic Characteristics

Participants' demographic data were collected, including gender, age, marital status, educational background, professional qualifications, occupational category, and department.

#### 12-Item General Health Questionnaire

The GHQ-12 is mainly used to evaluate the mental health of the general population and the existence of non-psychotic psychological-related states, and is widely used in the Chinese population (17, 18). The scale has undergone several rounds of revision, from 60 items to the present 12 items (including six forward-scores items and six reverse-scores items). Each item is rated on a four-point scale ranging from 0 to 3 representing “never,” “rarely,” “sometimes,” and “always,” respectively. The total score ranged from 0 to 36 points. The higher the total score, the lower the level of mental health. Further, a mean score of ≥2.25 indicated poor mental health. The scale was reasonably reliable in our sample, with a Cronbach's α of 0.871.

#### Goldberg Scales

The Italian version of the Goldberg Scale was used to assess anxiety and depression (19), which has been proven to have high reliability and validity in previous studies (20). The scale comprised nine items to assess anxiety and nine items for depression, with each item having a yes or no option. The response “yes” was scored as 1 point and “no” was scored 0. The total score on the anxiety and depression subscales ranged from 0 to 9. Individuals with an anxiety score of 5 or a depression score of 2 had a 50% chance of clinical manifestations; the probability increased sharply if the score was higher (21). Thus, an individual with a total standard score ≥ 5 was considered to have symptoms of anxiety, while those with a score ≥ 2 were considered to have symptoms of depression. The internal consistency coefficients of the anxiety and depression scales were 0.82 and 0.78, respectively.

### Data Analysis

The data were processed using Epidata and double-entered to ensure quality, and the characteristics of the data were analyzed using Mplus 7.0 and Stata 15.0. Descriptive statistics were performed for the demographic characteristics of participants, including quantity (n), percentage (%), mean.

The latent variable growth curve model (including the unconditional latent variable growth curve model and conditional latent variable growth curve model) was established based on the score of the GHQ-12. The load on the intercept factor was set to 1, and the four observation times were set as T1 = 0, T2 = 1, T3 = 2, and T4 = 3. The variable selected was the mean of the GHQ-12 score for each medical staff measured four times, and an unconditional latent variable growth curve model was established. Gender and type of violence (intimidation and physical violence) were used as control variables to establish the conditional latent variable growth curve model. Similarly, the trajectories of depression and anxiety for the medical staff at the four observation points were established. The normal distribution of continuous variables was verified by P-P diagram and K-S tests. CFI, TLI >0.90, and RMSEA <0.08 were used as the standard of the model fit index.

## Results

### Demographic Characteristics of the Respondents

Table 1 presents the demographic characteristics of the respondents. Medical staff suffered physical violence, accounting for 32.14% of the total. More than half of the participants were women (64.29%). A higher percentage of participants were below 30 years of age (50%). Most participants were undergraduates and above and 40.18% had worked for ≤5 years.

**Table 1:** Demographic characteristics of the respondents.

**Variables**	***n***	**%**
**Gender**		
Male	40	35.71
Female	72	64.29
**Age (years)**		
≤ 30	56	50.00
31–50	46	41.07
≥51	10	8.93
**Education**		
Below the junior college	31	27.68
Undergraduate	44	39.29
Master's and above	37	33.03
**Marital status**		
Married	68	60.71
Single/widowed/divorced	44	39.29
**Occupational category**		
Physicians	50	44.64
Nurses	62	55.36
**Professional titles**		
Primary title	56	50.00
Intermediate title	36	32.14
Senior title	20	17.86
**Department**		
Emergency department	16	14.29
Internal medicine	37	33.04
Surgery	22	19.64
Obstetrics and gynecology	3	2.68
Pediatrics	7	6.25
Eyes, nose and throat	6	5.36
Other	21	18.74
**Length of employment (years)**		
≤ 5	45	40.18
6–15	27	31.25
16–25	19	16.96
≥26	13	11.61
**Time of daily contact with patients (hours)**		
<4	16	14.29
4–8 h	39	34.82
>8	57	50.89
**Types of violence**		
Intimidation, threat	76	67.86
physical violence	36	32.14

### Medical Staff's Mental Health Loss Caused by Violence in the Workplace

#### The Incidence of General Health Status, Anxiety, and Depression Among Medical Staff at Different Observation Times

After the medical staff were subjected to intimidation, threats, and physical violence, their mental health status was 25.00, 52.68, 51.79, and 60.71% within 7 days, 1, 3, and 6 months, respectively. There was a significant difference in the incidence of psychological distress among medical staff at different observation times (*X*^2^ = 32.605, *P* < 0.001). The incidence of anxiety within 7 days, 1, 3, and 6 months was 29.46, 41.96, 45.54, and 49.11%, respectively. There was a statistically significant difference in the incidence of anxiety among medical staff at various observation times (*X*^2^ =10.112, *P* = 0.018). The incidence of depression within 7 days, 1, 3, and 6 months was 50.89, 50.89, 47.32, and 58.04%, respectively. There was no significant difference in the incidence of depression among medical staff at different observation times (*X*^2^= 2.718, *P* = 0.437).

#### The Changing Trajectory of the Mental Health Status of Medical Staff

**Figure 2:**
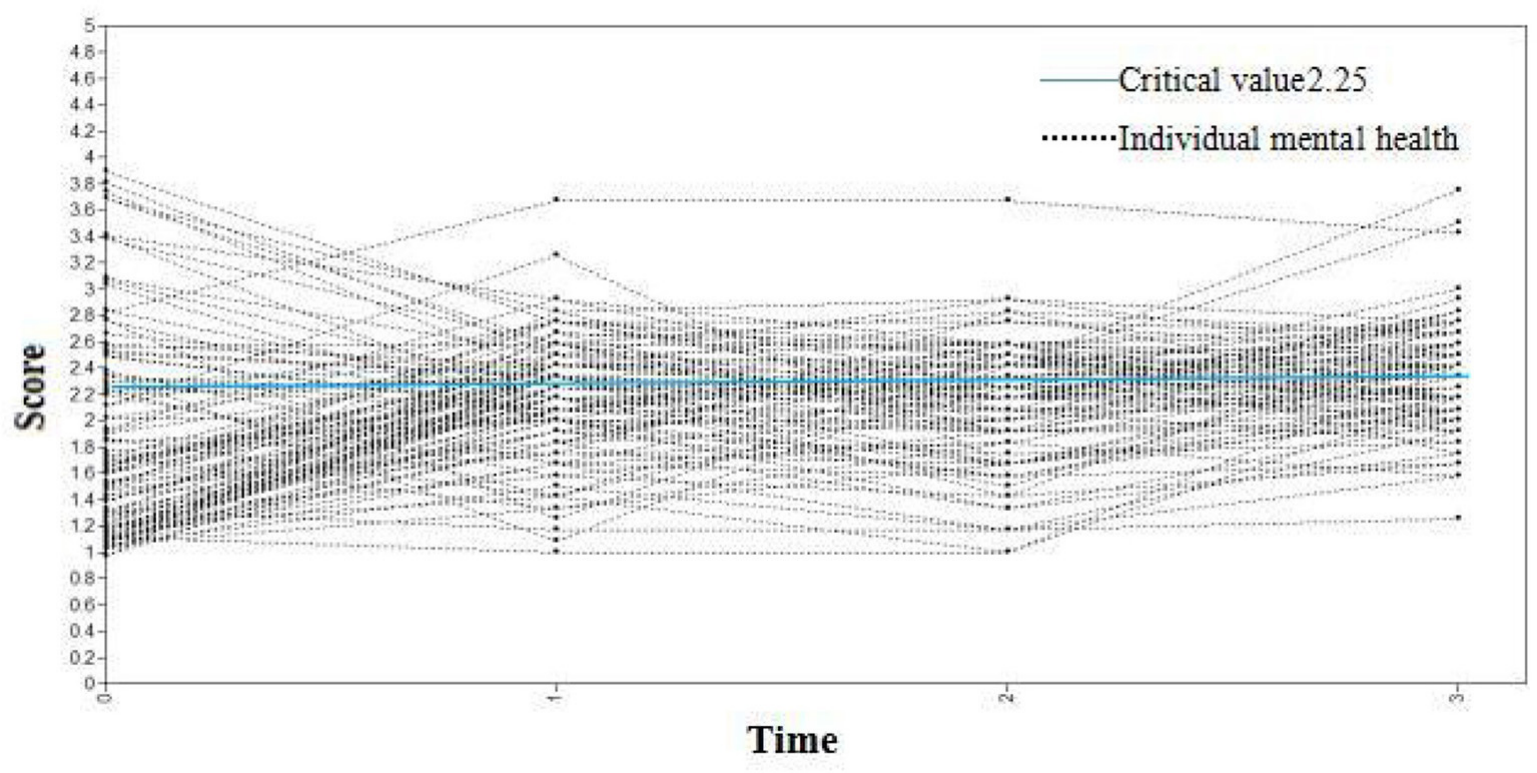
The changing trajectory of mental health status of medical staff at four different observation times.

Taking the score of the GHQ-12 of medical staff at four observation time points (7 days, 1, 3, and 6 months) as the dependent variable, an unconditional latent variable growth curve model was constructed. The fit indices of the model were: TLI = 0.914, CFI = 0.908, and RMSEA = 0.057. This shows that the model fit was acceptable. The variance estimates of the intercept and slope factors were 3.753 and 2.786, respectively, with P values < 0.001, indicating that there were significant individual differences in the initial general health level and growth rate of each medical staff. Figure 2 shows the variation curves of each medical staff's mental health at the four observation points. The light blue line indicates the critical value of mental health status is 2.25. The general health questionnaire scores of medical staff at each observation point ≥ 2.25 indicated that their mental health status was poor.

**Figure 3:**
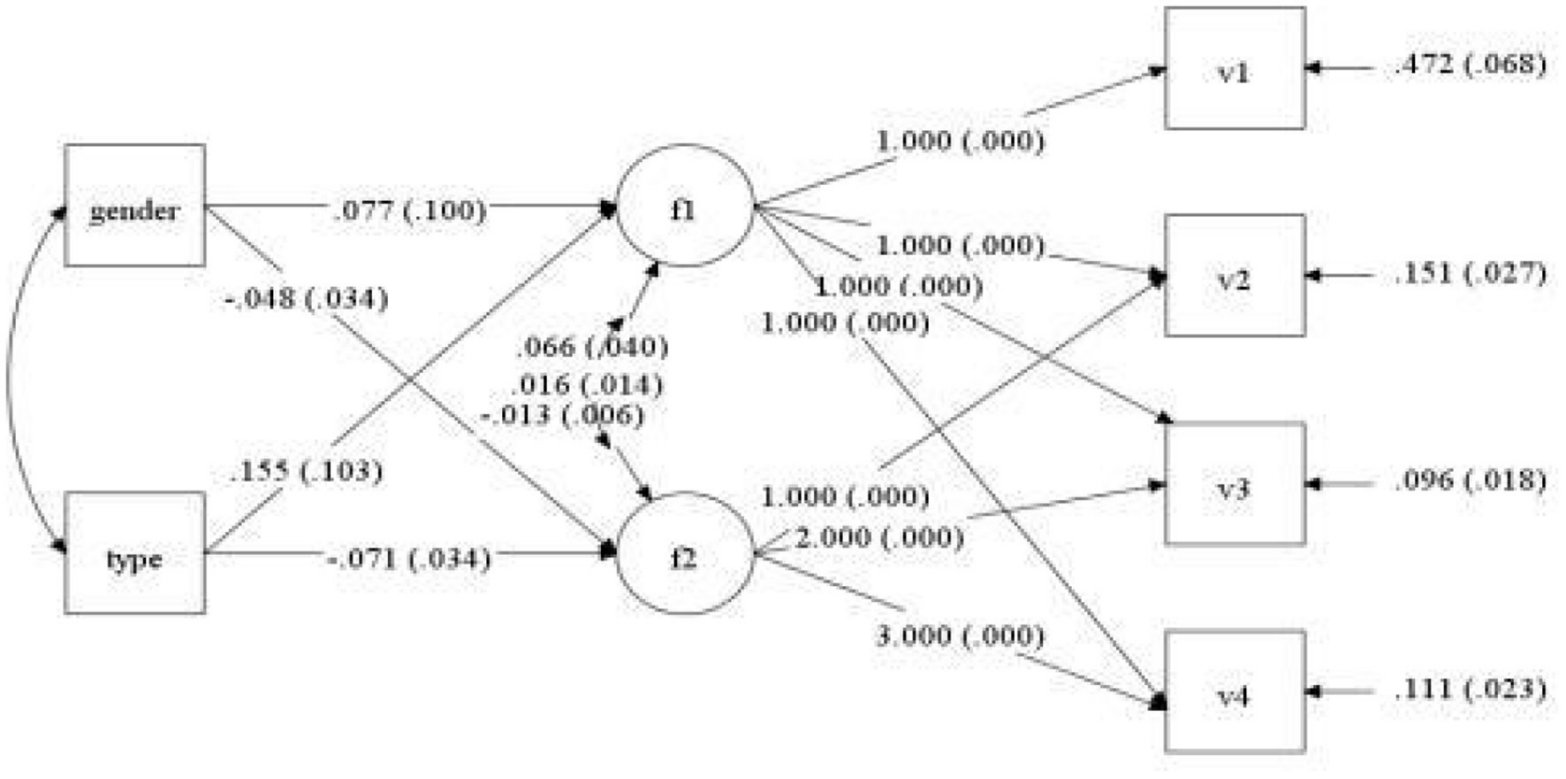
Path of latent variable growth curve model of mental health status of medical staff.

**Figure 4:**
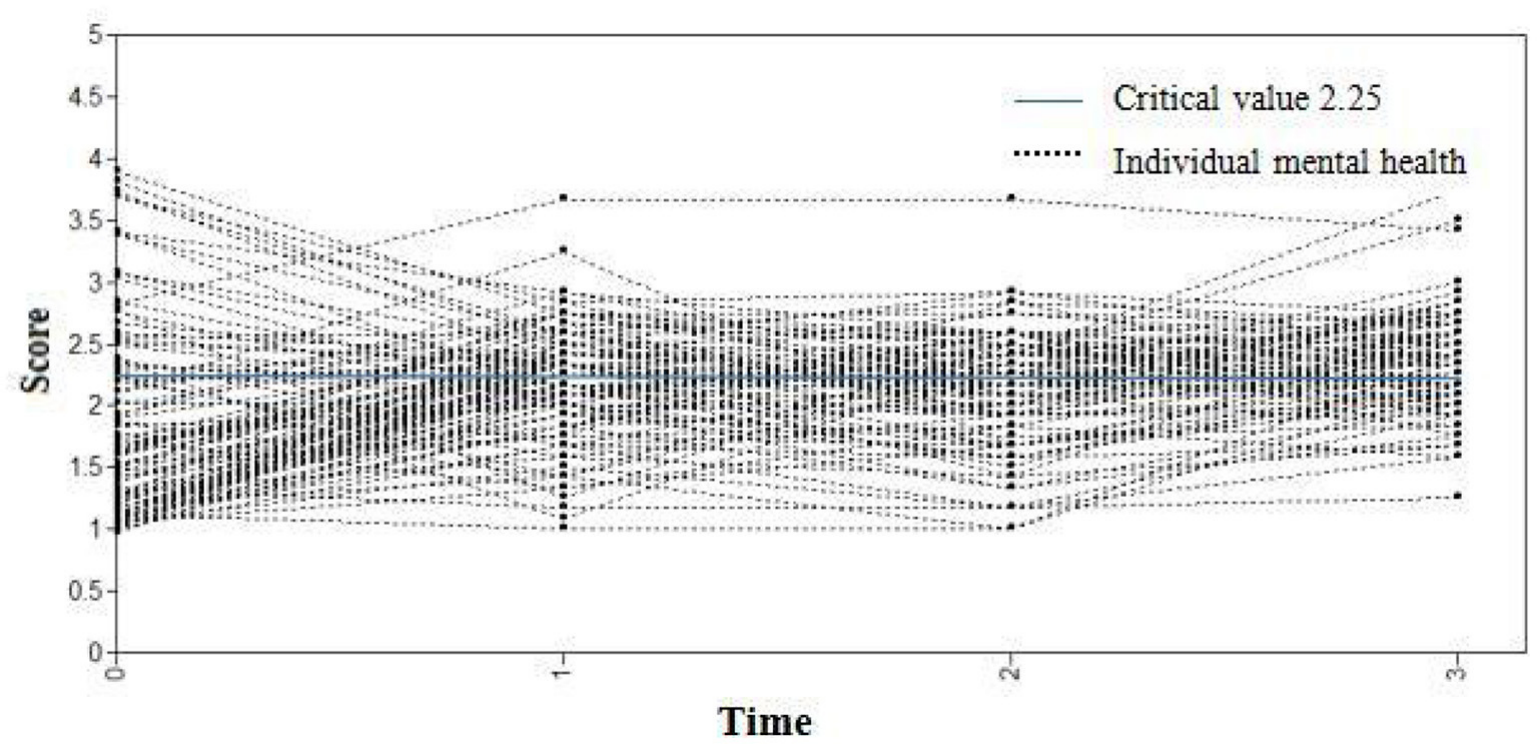
The changing trajectory of mental health level of medical staff after adjustment.

Gender and type of violence were employed as control variables, and mental health scores of medical staff at four observation points (7 days, 1, 3, and 6 months) were regarded as dependent variables to set up the conditional latent variable growth curve model. The fit indices of the model were TLI = 0.943, CFI = 0.926, and RMSEA = 0.051. This indicates that the model fit was acceptable, as shown in Figure 3. The regression coefficients of gender to slope and intercept factor were−0.048 (*P* = 0.157) and 0.077 (*P* = 0.445), respectively. It is suggested that there were no significant differences in the initial mental health level and mental health variation among medical staff of different genders. The regression coefficients of violence types on slope and intercept factors were−0.071 (*P* = 0.039) and 0.155 (*P* = 0.132), respectively. It was demonstrated that there was a difference in the initial mental health level of medical staff exposed to different types of violence, but there was no significant difference in the slope of mental health change. After the addition of control variables, the change curve of the mental health of each medical staff at four observation time points is displayed in Figure 4. The light blue line represents the critical value of mental health status 2.25. The score of the general health questionnaire of medical staff at each observation point ≥ 2.25 signified that their mental health status was poor.

#### The Changing Trajectory of Anxiety of Medical Staff

**Figure 5:**
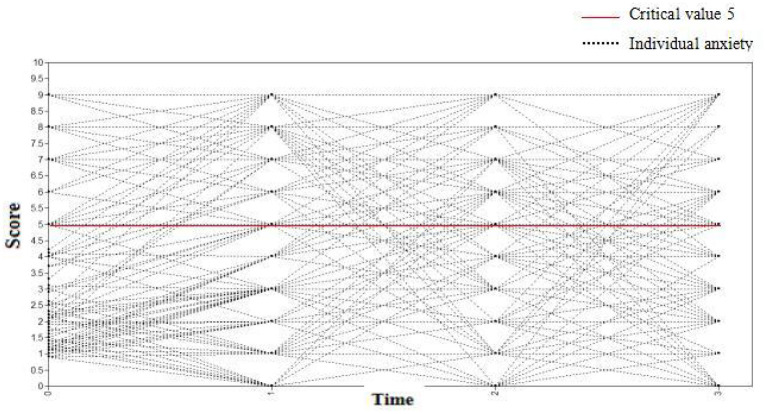
The changing trajectory of anxiety of medical staff at four observation time.

The anxiety scores of medical staff at four observation points (7 days, 1, 3, and 6 months) were selected as dependent variables to establish the unconditional latent variable growth curve model. The fit indices of the model were TLI = 0.907, CFI = 0.923, and RMSEA = 0.047. It was determined that the model fitted well. The variance estimates of the intercept and slope factors were 3.344 and 0.412, respectively, with P values < 0.001. It was believed that there were significant inter-individual differences in the initial anxiety level and growth rate of the medical staff. The correlation coefficient between the intercept growth factor and slope growth factor was−0.976 (*P* = 0.004). This demonstrated that individuals with high initial anxiety scores had a slower decline rate. As shown in Figure 5, an anxiety score ≥5 substantiated that medical staff had anxiety symptoms.

**Figure 6:**
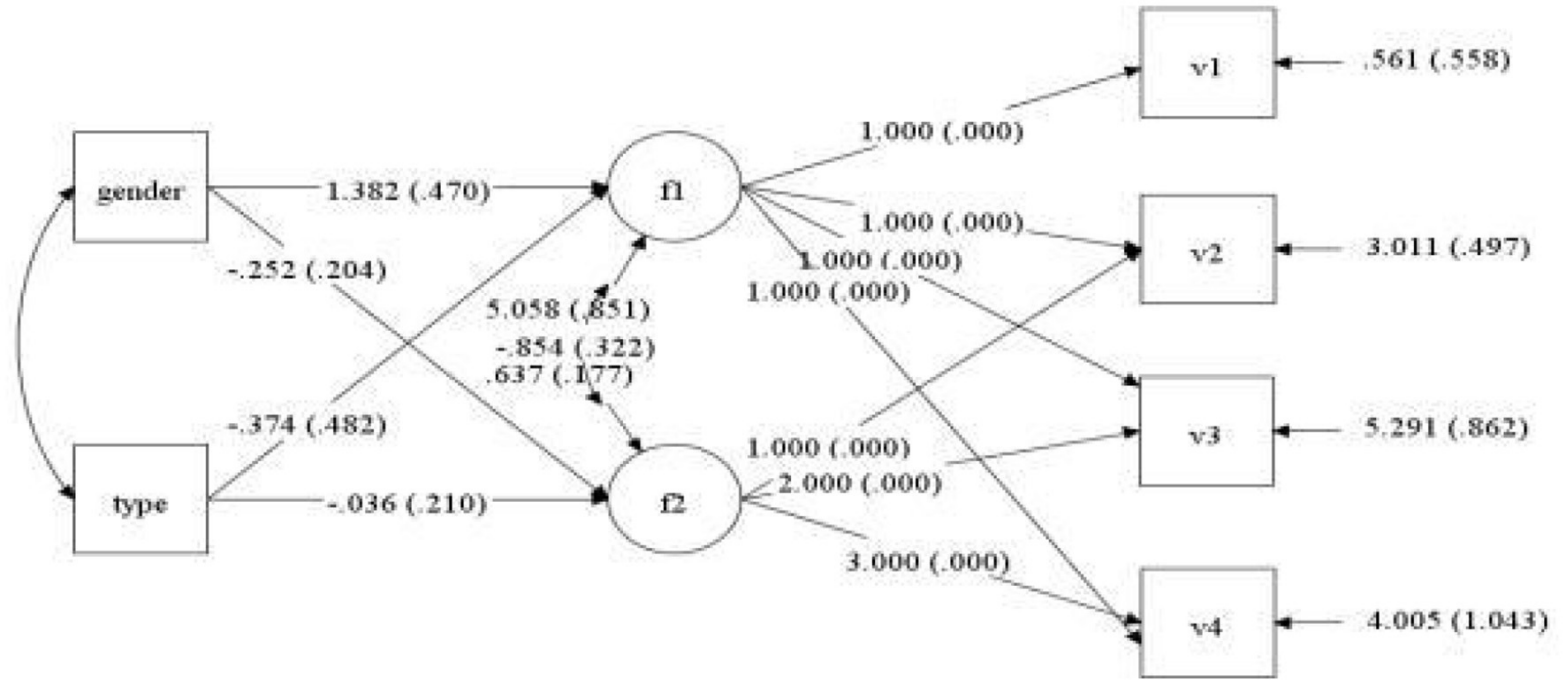
Path of latent variable growth curve model of anxiety of medical staff.

**Figure 7:**
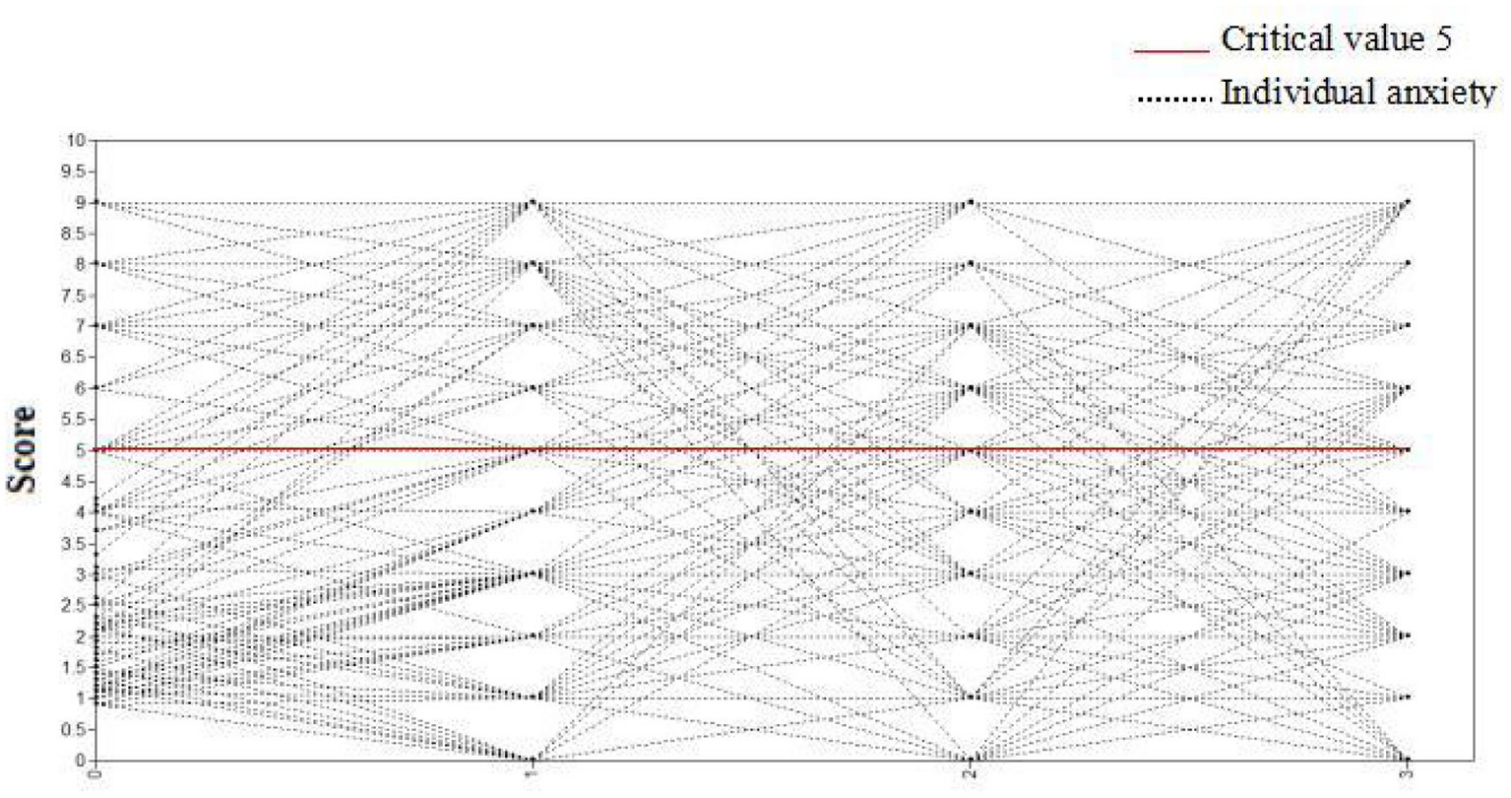
The changing trajectory of anxiety of medical staff after adjustment.

Gender and type of violence were employed as control variables, and anxiety scores of medical staff at four observation points (7 days, 1, 3, and 6 months) were selected as dependent variables to establish the conditional latent variable growth curve model. The fitting indices of the model were TLI = 0.950, CFI = 0.904, and RMSEA = 0.042. As such, the model fitted well, as shown in Figure 6. The correlation coefficient between intercept growth factor and slope growth factor was−0.854 (*P* = 0.008), indicating that individuals with high initial anxiety scores had a slower decline rate. The regression coefficients of gender on slope and intercept factors were−0.252 (*P* = 0.216) and 1.382 (*P* = 0.003), respectively. It should be noted here that there was no significant difference in initial anxiety state among medical staff of different genders, but there was a significant difference in the slope of anxiety state change. The regression coefficients of violence types on slope and intercept factors were−0.036 (*P* = 0.866) and−0.374 (*P* = 0.438), respectively. There was no significant difference in the initial anxiety level and the slope of anxiety state change after the medical staff suffered different types of violence. After the addition of control variables, the change curve of the anxiety state of each medical staff member is shown in Figure 7. An anxiety state score ≥5 for medical staff at each observation point indicated that they had an anxiety state.

#### The Changing Trajectory of Depression Among Medical Staff

**Figure 8:**
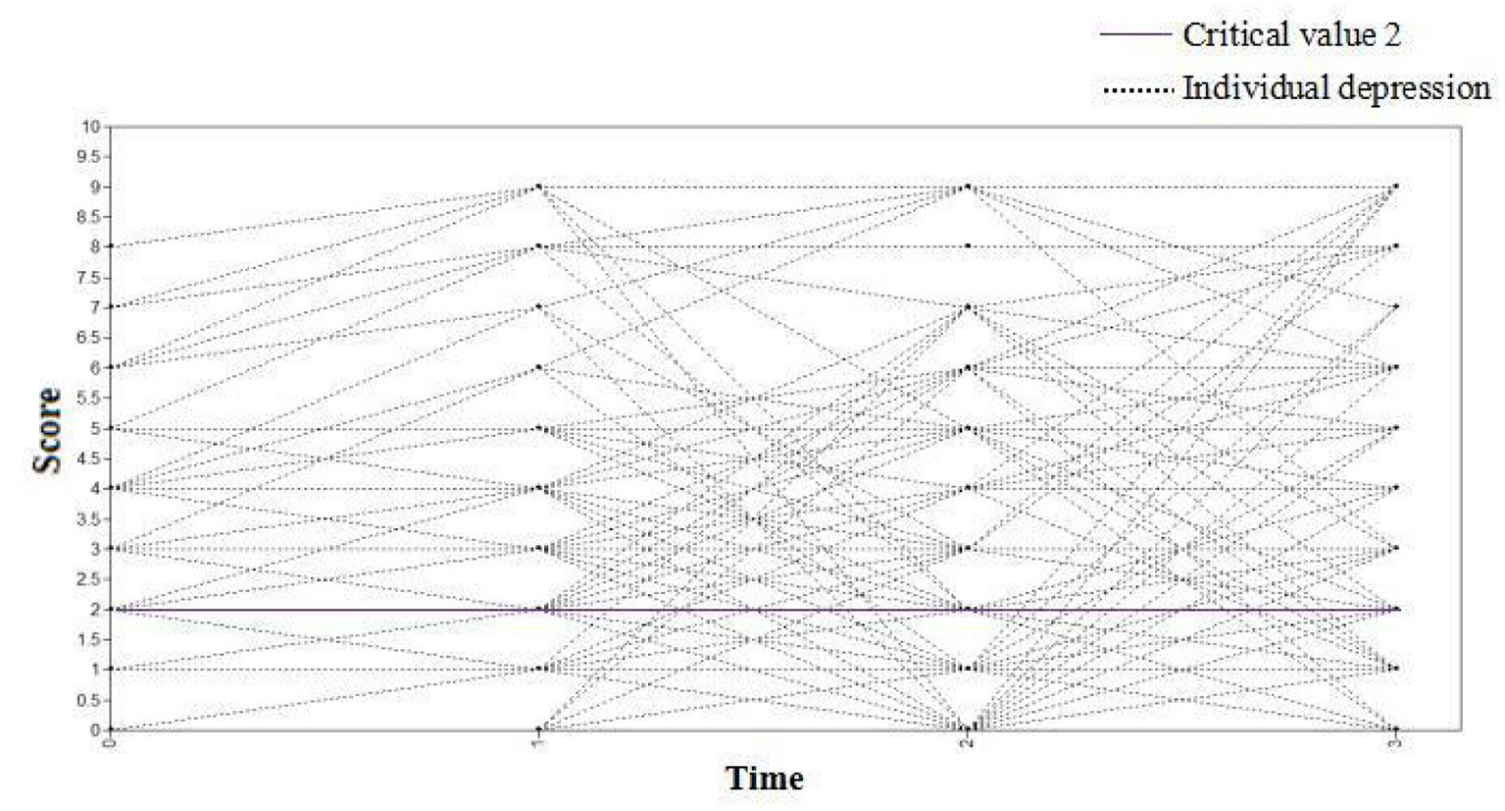
The changing trajectory of depression of medical staff.

The depression scores of medical staff at four observation times (7 days, 1, 3, and 6 months) were used as dependent variables to set up the unconditional latent variable growth curve model. The fitting indices of the model were TLI = 0.927, CFI = 0.939, and RMSEA = 0.037. This shows that the model fits well. The variance estimates of the intercept and slope factors were 4.934 and 0.561, respectively, with *P* values < 0.001. The results suggested that there were significant inter-individual differences in the initial depression level and growth rate of the medical staff. The correlation coefficient between the intercept growth factor and the slope growth factor was −0.854 (*P* < 0.001). It is noteworthy that individuals with high initial depression scores declined more slowly. Figure 8 shows the change curve of the depression status of each medical staff at the four observation points. The purple line indicates that the critical value of depression was 2, and the score of depression status ≥2 of medical staff at each observation point indicated that they were depressed.

**Figure 9:**
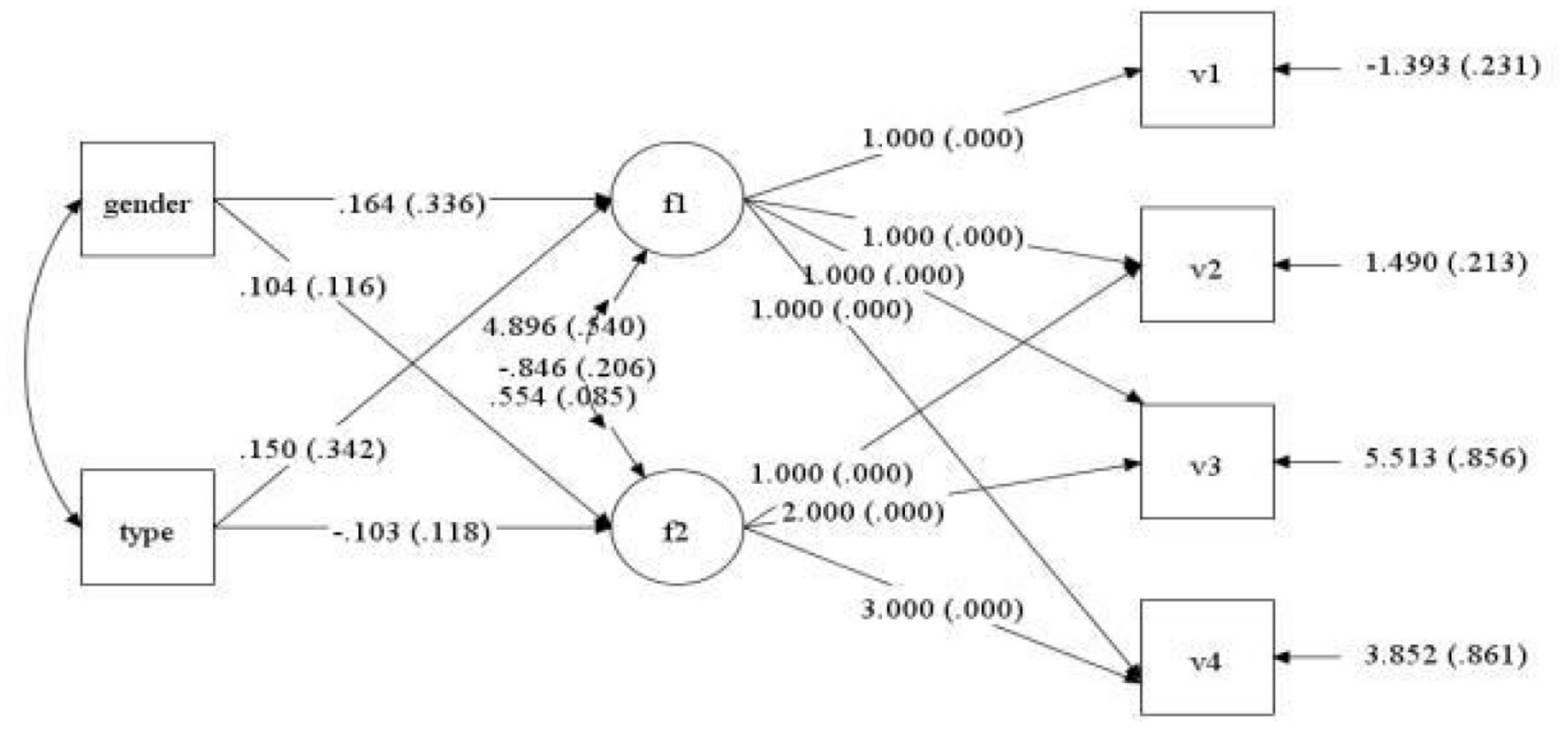
Path of latent variable growth curve model of depression in the medical staff.

**Figure 10:**
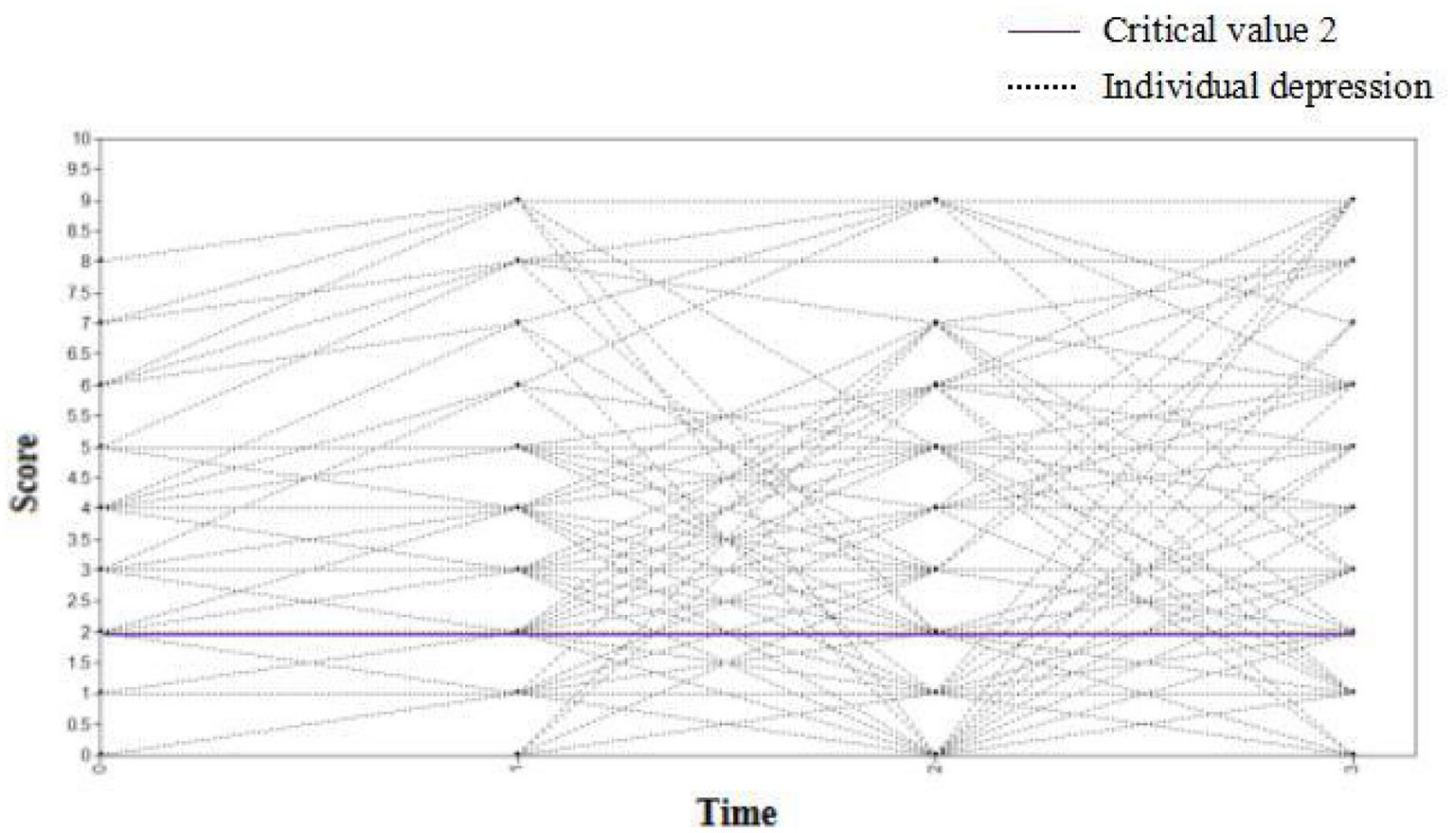
The changing trajectory of depression of the medical staff after adjustment.

Gender and type of violence were presented as control variables. Depression scores of medical staff at four observation points (7 days, 1, 3, and 6 months) were recorded as dependent variables to establish a conditional latent variable growth curve model. The fit indices of the model were TLI = 0.902, CFI = 0.931, and RMSEA = 0.044, indicating that the model fits well (Figure 9). The correlation coefficient between the intercept growth factor and the slope growth factor was−0.846 (*P* < 0.001), which indicated that individuals with high initial depression scores declined more slowly. The regression coefficients of gender to slope and intercept factors were 0.104 (*P* = 0.372) and 0.164 (*P* = 0.625), respectively. The results demonstrated no significant difference in the initial depression level and the slope of depression change among medical staff of different genders. The regression coefficients of violence type on slope and intercept factor were−0.103 (*P* = 0.383) and 0.150 (*P* = 0.662), respectively. However, it should be noted that there was no significant difference in the initial depression level and slope of depression after the medical staff experienced different types of violence. After the addition of control variables, the change in the curve of the depression status of each medical staff at the four observation points is shown in Figure 10. The purple line suggests that the critical value of depression was 2, and the score of depression status ≥ 2 of medical staff at each observation point indicated that they were depressed.

## Discussion

### Mental Health Loss of Medical Staff Owing to Workplace Violence

#### Incidence of Poor Mental Health, Anxiety, and Depression Among Medical Staff

The results revealed significant differences in the incidence of mental health, anxiety, and depression among medical staff at various observation times. We found that being intimidated, threatened, and physical violence have different effects on the mental health of medical staff in different time periods. Simultaneously, the results revealed that while medical staff's initial mental health indicators (mental health status, depression, and anxiety state) were better, the condition of linked indicators increased with time. This phenomenon could be because medical staff may receive prompt and substantial social support, such as from their bosses, co-workers, and families, following the incidence of workplace violence. Medical personnel benefited from the warmth of their families and organization, as well as received psychological comfort, and their mental health also improved (22–24). However, their organizational support returned to normal levels, the focus of family support shifted, and the incidence of poor mental health, anxiety, and depression increased with time. Another possibility is that the mental health of some medical staff changes slowly, and they are in the incubation period in the early stage, which gradually emerges over time. This may also be a reason for the rebound of mental health-related indicators.

There were significant differences between mental health at different observation times. This may be due to the greater changes in the incidence of mental health and anxiety state of medical staff after being threatened or experiencing physical violence. This indicated the need for sustained attention to the mental health of medical staff who have experienced violence in the hospital.

#### Changes in the Mental Health Index of Medical Staff

The results revealed significant individual differences in initial mental health, anxiety, depression, and growth rates of each of these states among medical staff. The possible reasons for this phenomenon are as follows: First, there were differences in the personality of each medical staff, which may lead to differences in the state and growth rate of their mental health indicators (25). Second, after facing workplace violence in hospitals, medical staff may implement various coping measures, such as positive coping and negative coping. These two coping styles could also cause differences in the initial mental health status of the medical staff (26, 27). Third, the medical staff had different social resources. Medical staff with wider social networks and stronger social capital could obtain more support from all aspects, making them face workplace violence in hospitals positively, even alleviate or eliminate their negative mental health status. Finally, there were differences in mental endurance or mental resilience among medical staff, leading to distinctions in mental health levels and growth rates of medical staff (25).

This study also revealed that at the initial assessment, participants had high anxiety and depression, but the alleviation of anxiety and depression was slow. This result may be because the medical staff adopted a negative response to violence in the hospital workplace, they have low utilization of social support. It may also be related to their personalities and, more likely, to a confluence of causes. It was found that positive communication with others after the occurrence of workplace violence in hospitals was conducive to the elimination of negative emotions and the acceleration of better mental health outcomes. Conversely, not accepting help and support from others may be conducive to worsening of mental health outcomes.

### Limitations

This study has a few limitations. In this dynamic study, owing to limited time and manpower and the difficulty in sample acquisition, only part of the third-class hospitals in two provinces was selected as the population, posing regional restrictions in terms of generalization. In the future, a large-scale national sample study is needed to verify the existing research results.

## Conclusion

At different observation times, there were significant disparities in the general health state of medical staff, as well as in the incidence of anxiety and depression. There were also considerable individual differences among medical staff in terms of baseline mental health, anxiety, depression, and growth rate of anxiety and depression. Therefore, hospitals should develop various psychological intervention strategies based on different stages of mental health to safeguard the wellbeing of medical staff who have experienced workplace violence.

## Data Availability Statement

The data that support the findings of this study are available from the corresponding author upon reasonable request.

## Ethics Statement

The study was approved by the Ethics Committee of the School of Public Health of Harbin Medical University (Project Identifier Code: HMUIRB20180305). We obtained the consent of each hospital involved in the research process. All participants gave informed consent to the researchers before the survey, and participants' personal information was kept confidential. The patients/participants provided their written informed consent to participate in this study.

## Author Contributions

LW, XN, LF, and LS conceived of and designed the experiments. LF and LS performed the experiments and critically revised the paper. LW and XN analyzed the data and wrote the paper. XN, YM, and WY contributed reagents/materials/tools of analysis. YZ and ZZ provided technical support. LG and XL reviewed and made substantial contribution to revision of the first draft. All authors revised the manuscript critically and approved the final version for publication.

## Funding

This study was funded by the National Science Foundation of China (Nos. 71473063 and 71874043), Directive Project of Medical Scientific Research Foundation in Guangdong (No. A2022379), Guangdong Basic and Applied Basic Research Foundation (No. 2020A1515110369), and Project funded by China Postdoctoral Science Foundation (No. 2021M701592). The funding body had no role in the design of the study, the collection, analysis and interpretation of the data, or writing of the manuscript.

## Conflict of Interest

The authors declare that the research was conducted in the absence of any commercial or financial relationships that could be construed as a potential conflict of interest.

## Publisher's Note

All claims expressed in this article are solely those of the authors and do not necessarily represent those of their affiliated organizations, or those of the publisher, the editors and the reviewers. Any product that may be evaluated in this article, or claim that may be made by its manufacturer, is not guaranteed or endorsed by the publisher.
